# Apple Origin Classification and Sugar Content Prediction of ‘Fuji’ Apples Using Near-Infrared Spectroscopy and Deep Learning

**DOI:** 10.3390/foods15122227

**Published:** 2026-06-20

**Authors:** Zhanglei Yan, Zhiyang Li, Zhihui Tang, Zhao Zhang, Tuanjie Li, Xuping Feng, Jingming Wu, Qu Xie, Xiaobo Li, Xu Li

**Affiliations:** 1 Key Laboratory of Tarim Oasis Agriculture, Ministry of Education, College of Information Engineering, Tarim University, Alar 843300, China; 17761717805@163.com (Z.Y.);; 2 National-Local Joint Engineering Laboratory of High Efficiency and Superior-Quality Cultivation and Fruit Deep Processing Technology on Characteristic Fruit Trees, Tarim University, Alar 843300, China; 3 Modern Agricultural Engineering Key Laboratory, Education Department of Xinjiang Uygur Autonomous Region, Alar 843300, China; 4 Key Laboratory of Smart Agriculture System Integration, Ministry of Education, China Agricultural University, Beijing 100083, China; 5 School of Mechano-Electronic Engineering, Xidian University, Xi’an 710071, China; 6 College of Biosystems Engineering and Food Science, Zhejiang University, Hangzhou 310058, China

**Keywords:** Fuji apple, near-infrared spectroscopy, origin classification, soluble solid content, °Brix prediction, deep learning

## Abstract

Accurate apple origin identification and non-destructive internal quality evaluation are important for fruit traceability, quality grading, and post-harvest management. Unlike previous studies mainly focusing on origin classification, this study established a dual-task near-infrared spectroscopy framework integrating geographical origin classification and soluble solid content (SSC, °Brix) prediction for Fuji apples. Samples were collected from three representative production regions in China: Alar in Xinjiang, Yantai in Shandong, and Luochuan in Shaanxi. Near-infrared diffuse reflectance spectra were acquired from 375 apples, generating 3000 spectral samples for origin classification and 750 SSC-calibrated samples for sugar content prediction. For classification, six deep learning models were evaluated using standardized full-spectrum input without chemometric spectral preprocessing, and the Transformer achieved the best performance, with a test accuracy of 96.22%. For SSC regression, spectra were preprocessed using standard normal variate and Savitzky–Golay filtering. The DNN model achieved the best prediction performance, with MAE = 0.5958 °Brix, RMSE = 0.7333 °Brix, R^2^ = 0.8646, and Pearson r = 0.9338. These results indicate that near-infrared spectroscopy combined with deep learning can support both Fuji apple origin authentication and non-destructive local tissue SSC assessment.

## 1. Introduction

Apple is one of the most important horticultural economic crops in China. Its industrial chain covers planting, post-harvest grading, storage, transportation, and processing, and plays an important role in rural employment and agricultural income growth. With the development of regional branding and high-value agricultural products, both origin labels and internal quality attributes of apples, such as soluble solid content (SSC, °Brix), have become important factors affecting market pricing, product circulation, and consumer acceptance. However, problems such as origin mislabeling and the sale of products with inconsistent quality may damage regional brands, reduce consumer trust, and increase the cost of market supervision and traceability. Therefore, the development of rapid, non-destructive, and objective methods for apple origin identification and internal quality evaluation is of practical importance for improving quality control, supporting origin traceability, and promoting the high-quality development of the apple industry.

In studies on apple origin classification, spectroscopy and imaging technologies combined with chemometric or machine learning methods have been widely investigated. Guo et al. [1] employed principal component analysis (PCA) and stepwise linear discriminant analysis (SLDA) to construct discriminant models for identifying apple juice varieties and geographical origins. Li et al. [2] used backpropagation neural networks, support vector machines, and extreme learning machines to distinguish apples from different varieties and regions, demonstrating the feasibility of near-infrared spectroscopy for origin identification. Eisenstecken et al. [3] proposed a rapid and non-destructive approach based on near-infrared spectroscopy combined with PCA-QDA, showing its potential for apple origin tracking and variety classification at the orchard scale. Luo et al. [4] further combined near-infrared spectroscopy with pattern recognition methods and proposed MWPLSDA, which enables the selection of informative narrow spectral bands while constructing high-precision classification models. These studies demonstrate that spectral information can effectively support apple origin identification. Nevertheless, challenges remain in improving discrimination among samples with similar spectral characteristics, enhancing model robustness under different batches and sampling conditions, and reducing dependence on complex manual feature engineering.

More recently, Cruz Sanchez et al. [5] reported a closely related study on the classification of apple growing regions using visible–near-infrared spectroscopy coupled with machine learning and deep learning methods. Their work investigated Chinese apple samples, including Fuji, Red Star, and Gala apples, from different growing regions and compared classification methods such as PLS-DA, SVM, and ANN-DA. The results further confirmed the potential of Vis–NIR spectroscopy combined with learning-based algorithms for apple growing-region classification, particularly for Fuji apples. However, most existing studies have mainly focused on origin or variety classification, whereas the integration of origin traceability with internal quality prediction remains relatively limited. In addition, further evaluation is still needed for end-to-end deep learning architectures using one-dimensional near-infrared spectra, especially when the application objective is extended from single classification modeling to both classification and quantitative prediction.

In the non-destructive prediction of fruit sugar content, usually represented by SSC, previous studies have mainly focused on spectral or imaging feature representation, the influence of measurement position, and quantitative modeling strategies. Mendoza et al. [6] fused key spectral features and image features from hyperspectral scattering images in the 500–1000 nm range, improving the prediction of apple firmness and SSC and showing that the integration of spectral and spatial structural information can enhance internal quality characterization. Fan et al. [7] collected spectral images from different apple positions, including the stem end, equator, and calyx, and systematically analyzed the influence of measurement position on SSC prediction. Their results indicated that differences in sampling position and fruit tissue structure can significantly affect spectral responses and modeling accuracy, highlighting the importance of standardized sampling strategies in practical applications. Mo et al. [8] combined near-infrared hyperspectral imaging with partial least squares regression (PLSR) to achieve visual mapping of SSC, providing a method for extending single-point prediction to spatial distribution evaluation. Lu et al. [9] explored the feasibility of multispectral imaging for quantifying fruit backscatter spectra to predict both firmness and SSC, showing potential for balancing detection efficiency and prediction accuracy. Although these studies have demonstrated the feasibility of non-destructive SSC prediction, model generalization under limited calibration samples, the reduction in destructive testing, and the comparative evaluation of deep learning models for different spectral analysis tasks still require further investigation.

Based on these considerations, this study investigated near-infrared spectroscopy for two complementary tasks in Fuji apples: geographical origin classification and non-destructive local tissue SSC prediction. A total of 375 Fuji apples were collected from three representative production regions in China: Alar, Xinjiang; Yantai, Shandong; and Luochuan, Shaanxi, with 125 apples obtained from each region. Six deep learning architectures, including 1D-ResNet, 1D-CNN, LSTM, Transformer, Dual-Stream, and DNN, were evaluated separately for the two tasks using task-specific input processing procedures, output layers, and optimization objectives. Traditional machine learning classifiers and PLSR were included only as reference baselines.

The main contribution of this study is the establishment and systematic evaluation of a dual-task near-infrared spectroscopy framework linking geographical origin traceability with local tissue SSC assessment. The contribution does not rely on any single network architecture; instead, it lies in examining how different architectural inductive biases perform across classification and quantitative prediction tasks using the same type of spectral information. This framework provides a broader technical basis for apple origin authentication, local quality evaluation, and post-harvest decision support.

## 2. Materials and Methods

### 2.1. Overview of Experimental Methods

**Figure 1:**
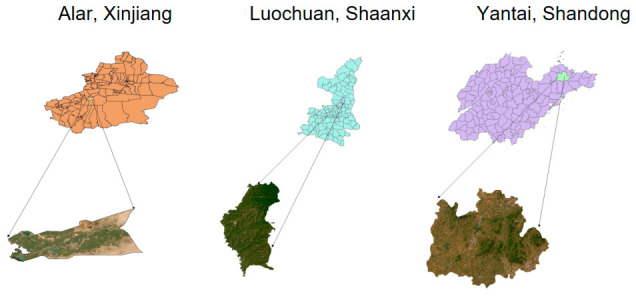
Sample Origin.

Fuji apples collected from three geographical regions in China—Alar in Xinjiang, Yantai in Shandong, and Luochuan in Shaanxi—were used in this study. Near-infrared diffuse reflectance spectroscopy was employed to acquire spectral information from the fruit surface, while a digital refractometer was used to determine the soluble solid content (SSC, °Brix) of tissue collected from the corresponding spectral sampling positions (Figure 1).

After spectral acquisition, the data were organized into one-dimensional spectral datasets for two complementary tasks: geographical origin classification and SSC regression. For origin classification, all spectral variables were retained and standardized using parameters estimated exclusively from the training set, without chemometric spectral preprocessing, manual wavelength selection, or handcrafted feature extraction. For SSC regression, the spectra were preprocessed using standard normal variate transformation and Savitzky–Golay first-derivative filtering, followed by feature standardization based on the training set.

Multiple deep learning architectures were then independently trained and evaluated for the two tasks. Task-specific output layers and loss functions were adopted for classification and regression, respectively, to assess the suitability of different network architectures for Fuji apple origin identification and non-destructive local tissue SSC prediction.

### 2.2. Fruit Samples

A total of 375 Fuji apples were collected from three production regions in China: Alar in Xinjiang, Yantai in Shandong, and Luochuan in Shaanxi, with 125 apples obtained from each region. All fruits were free from visible external defects. To minimize interference from dust, surface residues, and other contaminants during spectral acquisition, the fruit surfaces were gently wiped with a damp cloth and allowed to air-dry naturally before measurement.

Eight sampling points were then evenly marked around the equatorial region of each fruit, and one near-infrared spectrum was acquired at each marked position. Consequently, a total of 3000 one-dimensional near-infrared spectra were obtained for the geographical origin classification task (375 fruits × 8 sampling points per fruit).

The sugar content of the fruit was characterized by soluble solid content (SSC, °Brix). To reduce food waste caused by destructive sampling while ensuring calibration representativeness, 2 points were randomly selected from the 8 marked sampling points on each fruit for sugar content measurement. The sugar reference values at these points were paired with the corresponding near-infrared spectral data, resulting in a total of 750 sugar content samples (375 × 2).

To minimize the influence of environmental variation, all apple samples were equilibrated under the same indoor laboratory conditions prior to spectral acquisition and SSC measurement. The recorded laboratory temperature during sample equilibration, spectral acquisition, and SSC measurement was 28 °C. This temperature was the ambient laboratory temperature recorded during the experiment, rather than an intentionally selected representative temperature or a controlled temperature treatment. The spectral dataset was divided into training, validation, and test sets at a ratio of 70%/15%/15% using stratified random sampling by geographical origin to maintain similar class proportions across the three subsets.

All model training and data analysis were performed using Python 3.10 and PyTorch 2.5.0 on a computer equipped with an NVIDIA GeForce RTX 4060 Ti GPU (NVIDIA Corporation, Santa Clara, CA, USA) under Windows 10 (Microsoft Corporation, Redmond, WA, USA). The computing environment and main training parameters are summarized in Table 1.

### 2.3. Near-Infrared Spectroscopy Data Collection

A Thermo Scientific Antaris II Fourier transform near-infrared spectrometer (Thermo Fisher Scientific Inc., Waltham, MA, USA) was used to acquire near-infrared spectra from the Fuji apples. Spectral acquisition was conducted under consistent indoor laboratory conditions. Before measurement, the FT-NIR analyzer was switched on and allowed to operate under normal conditions for 30 min to stabilize the instrument response. No external heating device or additional heating procedure was used. After the warm-up period, the instrument self-check was completed, and a background reference spectrum was collected before each measurement batch.

**Figure 2:**
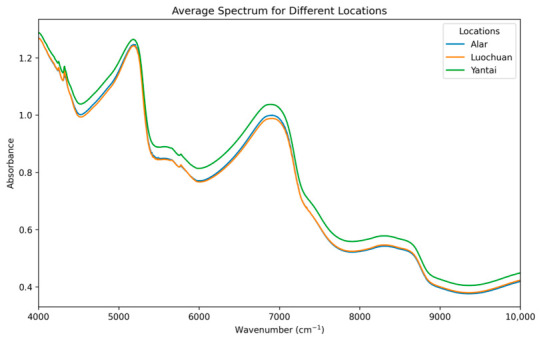
Average near-infrared spectra of Fuji apples from different production regions.

During spectral acquisition, an integrating-sphere accessory was used to obtain diffuse reflectance spectra from the fruit surface, and the spectra were recorded in absorbance mode. Under diffuse reflectance measurement, the detected spectral information mainly originated from the peel and shallow flesh tissue near the fruit surface rather than from the entire internal volume of the apple. The instrument parameters were set as follows: wavenumber range, 4000–10,000 cm^−1^; spectral resolution, 8 cm^−1^; number of scans, 32 per sampling position; and gain setting, 4. The 32 scans were averaged to improve the signal-to-noise ratio (Figure 2).

Although the instrument software was configured with a maximum background update interval of 24 h, this setting was not used as the actual interval between background measurements. Instead, a new background reference spectrum was collected before each measurement batch, and the spectral baseline was monitored throughout data acquisition to ensure measurement stability.

To ensure consistency among sampling positions, each fruit was sequentially measured at the marked equatorial positions while maintaining the same orientation relative to the integrating-sphere measurement window.

### 2.4. Measurement of Soluble Solid Content (SSC)

The soluble solid content (SSC, °Brix) of apples was measured using a WZB series digital refractometer (Shanghai INESA Scientific Instrument Co., Ltd., Shanghai, China). Before measurement, pure water was used for zero calibration, and the refractometer prism was cleaned and dried. According to the spectral sampling scheme, two points were randomly selected from the eight marked equatorial sampling points of each apple for SSC measurement. Fruit tissue was collected from the corresponding spectral sampling positions, and approximately 1 cm thick slices of fruit tissue were cut without peeling and pressed to extract juice into a beaker. Therefore, the SSC reference values used for regression modeling represented the local tissue SSC corresponding to the measured spectra, rather than the average SSC of the entire fruit.

The extracted juice was placed onto the refractometer prism for measurement. Measurements were taken every 10 s. When the readings stabilized and continuous fluctuations did not exceed 0.1 °Brix, the stable value was recorded as the SSC reference value for that sampling point. After each measurement, the prism was cleaned with pure water and dried to minimize residue effects on subsequent measurements. All SSC measurements were conducted under the same indoor laboratory conditions described in Section 2.2, with a recorded laboratory temperature of 28 °C, to ensure consistency among samples.

### 2.5. 1D-ResNet Model

Residual Networks (ResNet) were first proposed by He et al. [10] to address the “degradation problem” encountered in deep convolutional neural networks as the network depth increases. The degradation problem refers to the phenomenon where, with an increase in network depth, the model not only tends to overfit but also experiences an increase in training error. One of the main reasons for this is the significant difficulty in optimizing deep networks, where gradients tend to vanish during backpropagation, making it difficult for the model to converge effectively. To address this, ResNet introduces residual learning and skip connections, transforming the network’s learning goal from directly fitting the mapping H(x) to learning the residual F(x)=H(x)−x, so that the network output can be written as:

(1) y=F(x;θ)+x

In this context, x represents the input features, F(⋅) denotes the residual branch composed of several convolutional layers, and θ represents its parameters. This structure provides a more direct path for gradient propagation, significantly alleviating the instability issues in training deep networks, thereby supporting the construction of deeper networks with stronger expressive capabilities.

**Figure 3:**
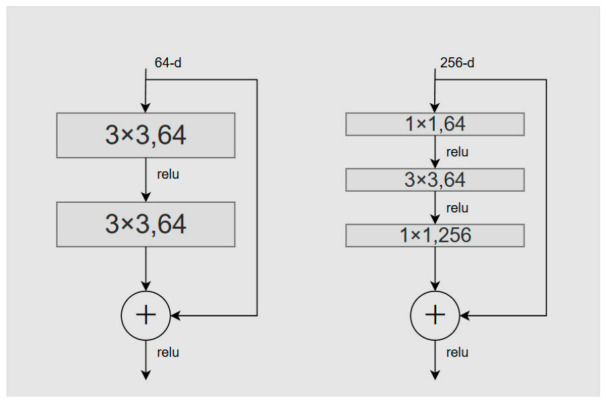
Residual Network Structure Diagram.

Near-infrared spectroscopy is a typical one-dimensional sequence data, with information arranged in wavenumber/wavelength order, containing local spectral segment shapes, absorption peaks, and their combined features. To apply the advantages of ResNet to spectral modeling tasks, researchers extended two-dimensional convolution and pooling operations to one-dimensional forms, forming 1D-ResNet. In 1D-ResNet, convolution kernels slide along the spectral dimension to extract local features from spectral segments, and residual connections are used to stabilize deep network training and enhance feature expression. For the basic residual block (BasicBlock), a common structure is a two-layer combination of Conv1D–BN–ReLU, followed by addition to the shortcut branch and then a nonlinear transformation. When the number of channels or sequence length changes (such as downsampling), a 1×1 Conv1D can be used to project the shortcut branch to match the dimensions. Based on this, 1D-ResNet is able to automatically extract discriminative spectral segment features from raw or lightly preprocessed spectra in an end-to-end learning framework, and has been widely used in one-dimensional spectral analysis tasks such as food quality detection and origin traceability (Figure 3).

### 2.6. Transformer Model

**Figure 4:**
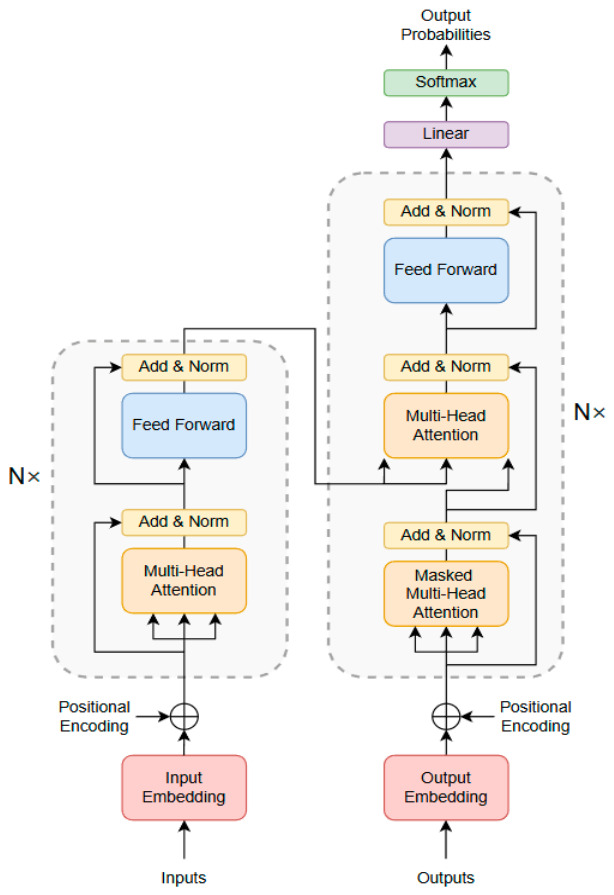
Overall Structure Diagram of Transformer.

The Transformer was originally introduced for natural language processing tasks [11,12,13,14,15,16,17,18]. Its key contribution lies in abandoning the traditional reliance on sequential computation in recurrent structures (such as RNN/LSTM) and instead employing the Self-Attention mechanism to model the relationships between different positions in a sequence globally. This approach enhances parallel computing efficiency and improves the model’s ability to capture long-range dependencies. For near-infrared spectroscopy, the spectral data are also a one-dimensional sequence signal arranged by wavenumber/wavelength, where complex correlations may exist between different spectral bands (e.g., coupling of multiple absorption bands, baseline drift, and local peak shape variations). Therefore, introducing Transformer into spectral analysis helps to learn the interaction relationships between spectral segments from a global perspective, improving feature extraction and discriminative ability (Figure 4).

In the Transformer model, the input sequence is first linearly mapped to obtain three representations: Query (Q), Key (K), and Value (V). The self-attention output is then computed using scaled dot-product attention:

(2) $$Attention(Q,K,V)=softmax(\frac{QK^{T}}{\sqrt{d_{k}}})V$$

In this context, $d_{k}$ represents the dimension of the key vector, which is used for scaling to stabilize the training process. This mechanism allows the model to weigh and aggregate sequence information based on relevance, enabling the model to automatically focus on the spectral features that are more important for the task, while suppressing redundant or noisy information.

To further enhance expressive capability, the Transformer employs Multi-Head Attention, which decomposes the attention mechanism into multiple subspaces for parallel learning:

(3) $$MHA(Q,K,V)=Concat(head_{1},\ldots ,head_{h})W^{o}$$

(4) $$head_{i}=Attention(QW_{i}^{Q},KW_{i}^{K},VW_{i}^{V})$$

In this context, h represents the number of attention heads, and $W_{i}^{Q}$, $W_{i}^{K}$, $W_{i}^{V}$, and $W^{o}$ are the learnable parameters. The multi-head structure allows the model to capture multi-scale associations between spectral segments from different subspaces, enhancing the model’s ability to model complex spectral patterns.

The Transformer encoder is typically composed of several stacked layers, each containing a multi-head self-attention module and a Feed-Forward Network (FFN), with residual connections and layer normalization to improve training stability. Since self-attention itself is insensitive to sequence order, the model typically requires the addition of positional encoding or learnable positional embeddings to preserve the order information of spectral points, thereby better representing peak positions, peak shapes, and spectral segment structures.

### 2.7. Dual-Stream Network Model

A near-infrared spectrum is a one-dimensional ordered signal arranged by wavenumber and contains information at different scales. Local spectral characteristics include absorption-peak shapes, peak widths, shoulders, and short-range variations, whereas broader spectral characteristics may involve baseline trends and relationships among separated absorption regions [19]. Depending on its architectural inductive bias, a single-branch network may emphasize either local patterns or long-range spectral relationships. Dual-stream architectures are therefore designed to integrate potentially complementary representations learned by two parallel feature-extraction branches, followed by a feature-fusion module. Common configurations include two convolutional branches with different kernel sizes or hybrid combinations of convolutional and sequential models, such as CNN–LSTM networks. However, combining multiple branches does not inherently guarantee improved predictive performance. The effectiveness of a dual-stream architecture depends on the complementarity of the learned features, the balance between the branches, the fusion strategy, model capacity, regularization, and optimization.

In this study, the Dual-Stream network consists of a 1D-ResNet branch and a Transformer branch. The 1D-ResNet branch uses one-dimensional convolutions and residual connections to stabilize deep feature learning, effectively extracting local spectral segment shapes and multi-scale convolution features. The Transformer branch models the global interaction relationships between spectral segments using the self-attention mechanism. Specifically, the Transformer branch first divides the one-dimensional spectrum into several patches of fixed length and obtains a token sequence through linear mapping; positional encoding is then added, and the data is fed into multiple encoder layers to obtain global representations, with the final sample-level feature vector obtained through CLS (or average pooling). The two branches output feature vectors $f_{cnn}$ and $f_{tr}$, which are fused by concatenation:

(5) $$f=[f_{cnn};f_{tr}]$$

This architecture was designed to integrate local convolutional representations with long-range attention-based spectral representations. Its effectiveness, however, depends on whether the two branches learn complementary features and whether the fusion module can be effectively optimized.

### 2.8. LSTM Model (With Attention Mechanism)

**Figure 5:**
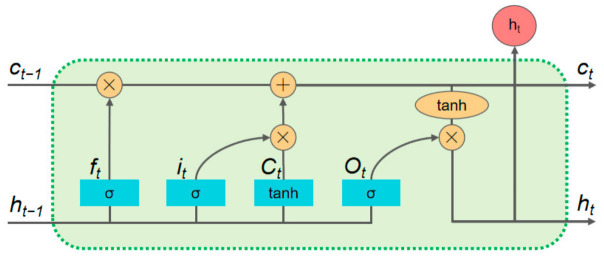
LSTM Framework Diagram.

Long Short-Term Memory (LSTM) is a classical variant of Recurrent Neural Networks (RNNs), initially introduced to address the gradient vanishing/explosion problem encountered in traditional RNNs when modeling long sequences [20,21,22,23,24,25,26]. LSTM incorporates a “memory cell” and gating mechanisms within the recurrent unit, allowing it to adaptively retain or forget historical information during sequence propagation, thereby modeling long-range dependencies more stably. For near-infrared spectroscopy, spectral points are arranged in wavenumber/wavelength order, essentially making the data a one-dimensional sequence. There are correlation patterns between different spectral bands, such as absorption peak coupling, baseline variations, and multi-peak combinations. Therefore, LSTM can theoretically be applied to learn the dependency structures and overall variation trends in the spectral sequence (Figure 5).

Using only the final hidden state of LSTM may not fully utilize the information from the entire sequence. To emphasize key spectral segments and enhance interpretability, this study introduces a temporal attention mechanism on the full sequence of hidden states $H=[h_{1},\ldots ,h_{T}]$ output by the LSTM, assigning different weights to different wavelength positions. The attention weights can be represented as:

(6) $$\alpha_{t}=softmax(s_{t})$$

(7) $$s_{t}=v^{T}tanh(Wh_{t}+b)$$

and obtain the context vector:

(8) $$c=\sum_{t=1}^{T}\alpha_{t}h_{t}$$

Here, $\alpha_{t}$ represents the contribution weight of the t-th spectral point (or spectral segment) to the final classification. This mechanism allows the model to automatically focus on spectral regions that are more sensitive to origin classification during the end-to-end training process, while suppressing redundant or noisy information.

### 2.9. DNN Model

**Figure 6:**
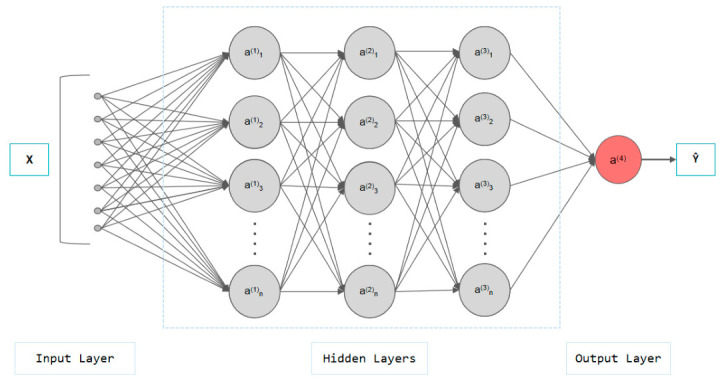
DNN Network Structure Diagram.

A deep neural network (DNN), also referred to as a multilayer perceptron, is a feedforward neural network composed of multiple fully connected layers [27,28,29,30]. Unlike convolutional or recurrent neural networks, a DNN treats each input spectrum as a fixed-dimensional feature vector and learns nonlinear relationships between the spectral variables and the prediction target through stacked linear transformations and nonlinear activation functions (Figure 6).

In this study, each near-infrared spectrum was represented as a one-dimensional vector containing all 1557 spectral variables. For geographical origin classification, the complete spectrum was used without SNV transformation, MSC, spectral smoothing, derivative transformation, manual wavelength selection, or handcrafted feature extraction. Each spectral variable was standardized using the mean and standard deviation estimated exclusively from the training set, and the same parameters were applied to the validation and test sets. For SSC regression, the spectra were preprocessed using SNV transformation and Savitzky–Golay first-derivative filtering, followed by feature standardization based on the training set, as described in Section 2.11.

The DNN used for geographical origin classification contained three hidden fully connected layers with 512, 256, and 128 neurons, respectively. Each hidden layer was followed by batch normalization, a GELU activation function, and dropout with a rate of 0.20. A three-neuron linear output layer was used to generate the logits corresponding to the three geographical origin classes. Therefore, the classification DNN contained three hidden fully connected layers and four fully connected layers in total, including the output layer. No convolutional or recurrent layers were included.

For SSC regression, a residual multilayer perceptron was used. The regression network contained four MLP blocks with output dimensions of 512, 512, 256, and 256, respectively. Each block contained two fully connected layers, resulting in eight hidden fully connected layers in total. The output dimensions of these hidden layers were 512, 512, 512, 512, 256, 256, 256, and 256, respectively. An input batch-normalization layer was applied before the MLP blocks. Within each block, the first fully connected layer was followed by GELU activation and dropout with a rate of 0.15, while the second fully connected layer was followed by batch normalization and GELU activation. A residual connection was used when the input and output dimensions of a block were identical. Finally, a single-neuron linear output layer generated the continuous SSC prediction. Thus, the regression DNN contained eight hidden fully connected layers and nine fully connected layers in total when the output layer was included. No convolutional or recurrent layers were used.

Let the input spectral vector be $x\in R^{L}$. The hidden representation at the l-th layer of the DNN can be written as:

(9) $$h^{\left(l\right)}=\phi (W^{\left(l\right)}h^{\left(l-1\right)}+b^{\left(l\right)})$$

Here, $h^{\left(l-1\right)}$ and $h^{\left(l\right)}$ denote the input and output representations of the l-th fully connected layer, respectively; $W^{\left(l\right)}$ and $b^{\left(l\right)}$ are the corresponding learnable weight matrix and bias vector; and ϕ denotes the nonlinear transformation. For geographical origin classification, the final linear layer generated three class logits, whereas for SSC regression, the final linear layer generated one continuous SSC value. The task-specific output interpretations and loss functions are described in Section 2.11.

### 2.10. 1D-CNN Model

One-dimensional convolutional neural networks (1D-CNNs) are designed for one-dimensional ordered signals and can automatically learn local representations through shared convolution kernels [31,32,33,34,35,36]. In this study, each near-infrared spectrum was represented as an ordered one-dimensional signal containing all 1557 spectral variables arranged by wavenumber.

For the geographical origin classification task, the 1D-CNN received the complete spectrum after variable-wise standardization using the mean and standard deviation estimated exclusively from the training set. The same standardization parameters were subsequently applied to the validation and test sets. No standard normal variate transformation, multiplicative scatter correction, spectral smoothing, derivative transformation, manual wavelength selection, or handcrafted feature extraction was applied before classification. Therefore, the expression “without manual feature engineering” refers to the automatic learning of spectral representations by the convolutional layers and does not imply that numerical standardization was omitted or that the input spectra were completely unprocessed. For the SSC regression task, the 1D-CNN received spectra preprocessed using standard normal variate transformation and Savitzky–Golay filtering with a window size of 11, a polynomial order of 3, and the first derivative, followed by feature standardization based exclusively on the training set, as described in Section 2.11.

The convolutional layers learned local spectral patterns, including absorption-peak shapes, shoulders, widths, and relative positions, directly from the input spectra. Pooling and downsampling operations progressively reduced the spectral resolution and generated increasingly abstract feature representations, while also improving robustness to local noise and small spectral variations. The resulting features were subsequently used by the task-specific output layer for geographical origin classification or SSC regression.

Let the input spectrum be $x\in R^{L}$. The one-dimensional convolution layer slides a convolution kernel W over the sequence, performing local weighted summation and outputting feature maps:

(10) $$y(t)=\sum_{i=1}^{k}w(i)x(t+i)+b$$

Here, k represents the size of the convolution kernel. A nonlinear activation function is typically applied after the convolution layer to enhance the model’s ability to express nonlinear relationships. Batch Normalization (BN) is also used to stabilize training and accelerate convergence, while Dropout is employed to prevent overfitting.

### 2.11. Task-Specific Implementation of the Dual-Task Framework

The dual-task framework in this study comprised two independently trained supervised learning tasks: geographical origin classification and SSC regression. It was not implemented as a jointly optimized multi-output network, and no parameters were shared between the two tasks during training. The same six backbone model families were evaluated separately for the two tasks, while their input processing procedures, output layers, target formats, and optimization objectives were adapted according to the task type.

For the geographical origin classification task, all models received the complete set of 1557 spectral variables. No chemometric spectral preprocessing, including standard normal variate (SNV) transformation, multiplicative scatter correction (MSC), spectral smoothing, or derivative transformation, was applied. In addition, no manual wavelength selection or handcrafted spectral feature extraction was performed. After the spectral samples were divided into training, validation, and test sets, each spectral variable was standardized using the mean and standard deviation estimated exclusively from the training set. The same standardization parameters were then applied to the validation and test sets. Therefore, the classification inputs are described as standardized full-spectrum data without chemometric spectral preprocessing or manual feature engineering, rather than as completely unprocessed raw spectra.

For the SSC regression task, all models received spectra preprocessed using SNV transformation and Savitzky–Golay first-derivative filtering. The Savitzky–Golay parameters were set to a window size of 11 and a polynomial order of 3. Feature standardization was subsequently performed using the mean and standard deviation estimated exclusively from the training set, and the same parameters were applied to the validation and test sets. The SSC reference values were also standardized using the training-set mean and standard deviation to improve numerical stability during model optimization. After prediction, the regression outputs were inverse-transformed to the original °Brix scale for performance evaluation.

For geographical origin classification, each backbone was connected to a three-neuron linear output layer corresponding to the three production regions. The output layer generated three unnormalized logits. During training, the models were optimized using the cross-entropy loss function. Softmax activation was not applied before calculation of the cross-entropy loss because the PyTorch implementation of cross-entropy directly operates on unnormalized logits. During inference, the softmax function was applied to convert the logits into class probabilities, and the class with the highest probability was selected as the predicted geographical origin.

For SSC regression, each backbone was connected to a single-neuron linear output layer that directly generated one continuous SSC value. No softmax or other output activation function was used, and the cross-entropy loss used for classification was not applied. Instead, the primary regression objective was the smooth L1 loss, also referred to as the Huber loss, with β = 1.0. A Pearson correlation auxiliary term was additionally introduced to encourage consistency between the variation trends of the predicted and reference SSC values. The total regression loss was defined as:

(11) $$L_{reg}=L_{Huber}+0.05(1-r)$$

where $L_{Huber}$ denotes the smooth L1 loss and r denotes the Pearson correlation coefficient between the predicted and reference SSC values within a training batch. The Huber-loss component constrained pointwise prediction errors while reducing sensitivity to relatively large residuals, whereas the correlation term encouraged agreement between the predicted and measured SSC trends.

Accordingly, the two tasks differed explicitly in their spectral preprocessing procedures, output dimensions, target formats, output interpretations, and loss functions. The expression “without manual feature engineering” used for origin classification refers specifically to the absence of manual wavelength selection and handcrafted spectral feature extraction; it does not imply that numerical standardization was omitted.

### 2.12. Evaluation Metrics

To comprehensively evaluate the performance of the model in apple origin classification and non-destructive SSC (soluble solid content) prediction tasks, classification metrics and regression metrics are used for quantitative assessment.

#### 2.12.1. Origin Classification Evaluation Metrics

Let there be N samples in the test set, and the number of classes be K. The true labels are denoted as $y_{i}\in \{1,\ldots ,K\}$, and the predicted labels are denoted as y^i. The element $C_{jk}$ of the confusion matrix is defined to represent the number of samples whose true class is j and predicted class is k. For class k, the following is defined:

(12) $$TP_{k}=C_{kk}\;(\mathrm{True}\;\mathrm{Positive})$$

(13) $$FP_{k}=\underset{j\neq k}{\sum }C_{jk}\;(\mathrm{False}\;\mathrm{Positive})$$

(14) $$FN_{k}=\underset{j\neq k}{\sum }C_{kj}\;(\mathrm{False}\;\mathrm{Negative})$$

1. Accuracy

Representing the proportion of overall correct predictions:

(15) Accuracy=1N∑i=1N∣(yi^=yi)

2. Precision/Recall/F1-score

It respectively measures the proportion of true samples among those predicted as this class, the proportion of correctly identified samples among all true samples of this class, and the harmonic mean of these two metrics:

(16) $$P_{k}=\frac{TP_{k}}{TP_{k}+FP_{k}}$$

(17) $$R_{k}=\frac{TP_{k}}{TP_{k}+FN_{k}}$$

(18) $$F1_{k}=\frac{2P_{k}R_{k}}{P_{k}+R_{k}}$$

3. Confusion Matrix

The confusion matrix is used to visualize the misclassification patterns among different producing areas. In addition to the count matrix, this paper also adopts the row-normalized form:

(19) $${\overset{\sim }{C}}_{jk}=\frac{C_{jk}}{\sum_{m=1}^{K}C_{jm}}$$

Here, ${\overset{\sim }{C}}_{jk}$ represents the proportion of samples that are true class j but predicted as class k.

#### 2.12.2. SSC Regression Evaluation Metrics

Let the true SSC in the test set be $y_{i}$, the predicted SSC be yi^, the sample size be N, and the true mean be $\overset{¯}{y}$:

(20) $$\overset{¯}{y}=\frac{1}{N}\sum_{i=1}^{N}y_{i}$$

1. MAE

It measures the magnitude of the average deviation and has relatively good robustness:

(21) MAE=1N∑i=1N|yi−yi^|

2. RMSE

It is more sensitive to large errors:

(22) RMSE=1N∑i=1N(yi−y^i)2

3. R^2^

It measures the model’s ability to explain the variance of the true values:

(23) R2=1−∑i=1N(yi−y^i)2∑i=1N(yi−y¯)2

4. Pearson

It measures the degree of linear correlation between the predicted values and the true values:

(24) r=∑i=1N(yi−y¯)(y^i−y^¯)∑i=1N(yi−y¯)2∑i=1N(y^i−y^¯)2

## 3. Results

This study evaluated near-infrared spectroscopy for two complementary tasks: three-class geographical origin classification and non-destructive local tissue SSC prediction of Fuji apples. The experimental settings, data partitioning strategy, task-specific input processing procedures, output layers, and optimization objectives followed those described in Section 2. For the origin classification task, all 1557 spectral variables were used as standardized full-spectrum input. No chemometric spectral preprocessing, such as SNV, MSC, smoothing, or derivative transformation, and no manual wavelength selection or handcrafted feature extraction were applied. The standardization parameters were estimated exclusively from the training set and subsequently applied to the validation and test sets. For the SSC regression task, the spectra were preprocessed using SNV transformation and Savitzky–Golay first-derivative filtering with a window size of 11 and a polynomial order of 3, followed by feature standardization based exclusively on the training set. Classification performance was evaluated using accuracy, precision, recall, F1-score, and confusion matrices, whereas regression performance was assessed using MAE, RMSE, R^2^, and the Pearson correlation coefficient. Traditional machine learning classifiers were included only as baseline references for the origin classification task, while PLSR was used as the chemometric baseline for SSC regression.

### 3.1. Origin Classification Results

**Figure 7:**
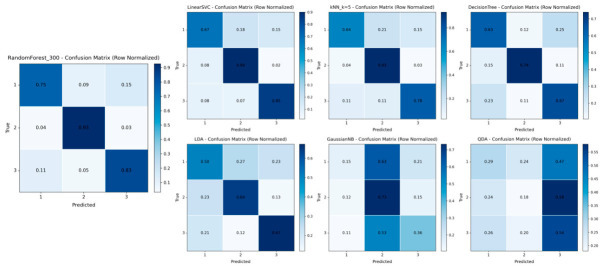
Normalized Confusion Matrix for Machine Learning.

**Figure A1:**
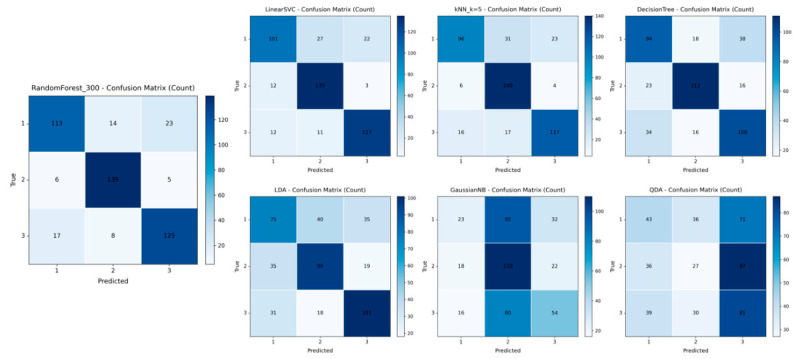
Original count-based confusion matrices of conventional machine learning classifiers.

**Figure A2:**
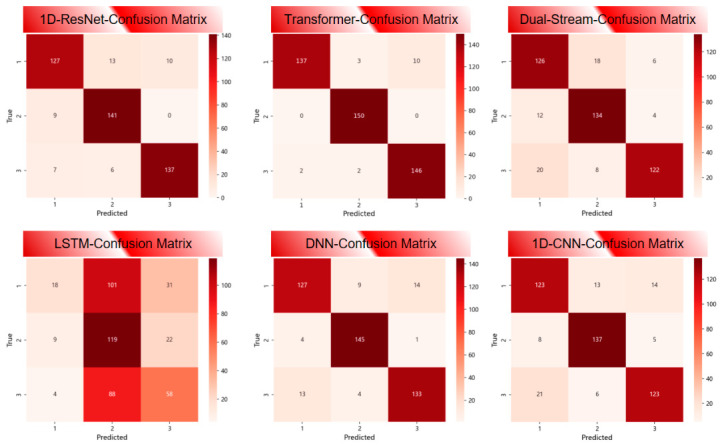
Original count-based confusion matrices of deep learning models.

Several conventional classifiers were evaluated only to provide contextual baseline values for the geographical origin classification task. Their test accuracies ranged from 0.3356 to 0.8378, with Random Forest achieving the highest baseline accuracy of 0.8378. Based on the row-normalized confusion matrix, Random Forest achieved recall values of approximately 0.75, 0.93, and 0.83 for Xinjiang, Luochuan, and Yantai, respectively. Because the principal objective of this study was to evaluate the task-dependent behavior of deep learning architectures within the proposed classification–regression framework, the conventional classifiers were not treated as the central comparison. The subsequent analysis therefore focuses on the six deep learning models. The original count-based confusion matrices of the conventional classifiers and deep learning models are provided in Appendix A as Figure A1 and Figure A2, respectively, to support data reproducibility, while the normalized confusion matrices are retained in the main text to facilitate class-wise performance comparison (Figure 7).

**Figure 8:**
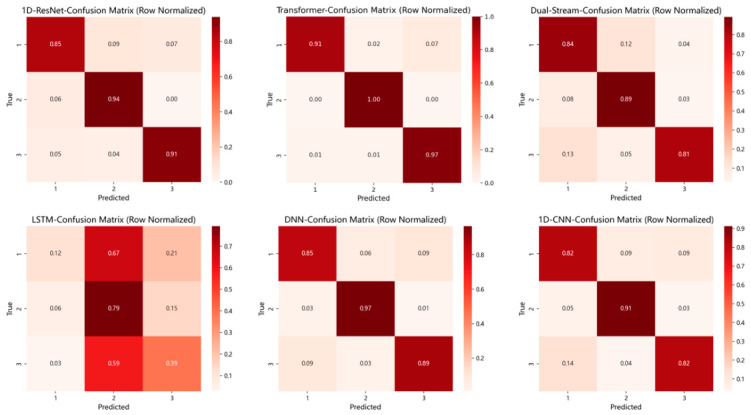
Normalized Confusion Matrix for Deep Learning.

The class-specific recall values of the deep learning models were also obtained from the row-normalized confusion matrices. The Transformer achieved recall values of approximately 0.91, 1.00, and 0.97 for Xinjiang, Luochuan, and Yantai, respectively. The corresponding recall values were approximately 0.85, 0.94, and 0.91 for the 1D-ResNet and 0.85, 0.97, and 0.89 for the DNN. These results indicate that the Transformer provided the most balanced class-specific performance among the evaluated models, while Luochuan apples were generally identified more accurately than the other two origins (Figure 8).

To facilitate the interpretation of the performance differences among deep learning models, the main architectural characteristics of each model are summarized below. These descriptions are intended to explain the observed classification behavior of different architectures, while the main focus of this study remains the evaluation of deep learning models within the proposed dual-task near-infrared spectroscopy framework.

For 1D-ResNet, a ResNet-18-style one-dimensional BasicBlock was adopted as the backbone. Each residual block consisted of two Conv1D-BN-ReLU layers. When the stride or number of channels changed, a 1×1 Conv1D + BN projection shortcut was used to match the feature dimensions. This residual design improved gradient propagation and enhanced the optimization stability of the deep network. The network contained four stages, with two residual blocks stacked in each stage. Downsampling with stride = 2 was applied between adjacent stages, and the number of channels was doubled to form multi-scale representations of spectral features. Finally, adaptive average pooling was used to compress the feature maps into fixed-dimensional vectors, and Dropout was introduced before the classification head to reduce overfitting.

For the Transformer, this study constructs a patch-token representation for one-dimensional spectra: the spectrum of length L is divided into patches with a length of 16 spectral points, and a linear mapping $R^{P}\to R^{d}$ is applied to obtain a token sequence (with zero padding for the remaining part if necessary). Subsequently, sinusoidal positional encoding is added, and the data is fed into a Transformer Encoder using Pre-LN (norm_first = True) to enhance training stability and the ability to model long-range spectral dependencies. Sample-level features are aggregated using the CLS-token pooling, followed by a linear classification head to output class logits. During training, AdamW, ReduceLROnPlateau, and Early Stopping are used to improve convergence stability and generalization performance.

In the Dual-Stream architecture, a 1D-ResNet branch and a Spectral Transformer branch were combined in parallel. The 1D-ResNet branch mainly extracted local spectral peak features and multi-scale convolutional representations, whereas the Transformer branch modeled global dependencies among spectral segments. The feature vectors from the two branches were fused for classification, allowing the model to integrate local and global spectral information.

For the LSTM model, a temporal attention aggregation mechanism was introduced to assign different weights to hidden states at different spectral positions. This design allowed the model to focus on spectral regions that were more relevant to origin classification, rather than simply averaging the entire sequence. A two-layer stacked LSTM structure with Dropout was used to improve representational capacity and reduce overfitting. The aggregated sequence features were then fed into a lightweight multilayer perceptron classification head.

For the DNN model, each near-infrared spectrum was treated as a fixed-dimensional global feature vector with 1557 input variables. The network consisted of three hidden fully connected layers containing 512, 256, and 128 neurons, respectively. Each hidden layer was followed by batch normalization, a GELU activation function, and dropout with a rate of 0.20. The final fully connected output layer contained three neurons corresponding to the three geographical origin classes. Therefore, the DNN contained three hidden fully connected layers and four fully connected layers in total when the output layer was included. No convolutional layers were used in this architecture. The DNN directly learned the nonlinear mapping from the full spectral vector to the origin classes without explicitly imposing local convolutional or sequential modeling assumptions.

For the 1D-CNN model, near-infrared spectra were treated as one-dimensional signals arranged along the wavelength axis. Convolutional layers were used to extract local spectral features, such as absorption peaks, shoulders, peak widths, and relative positions. The network adopted a hierarchical convolutional feature extraction strategy. A larger convolution kernel was first used in the stem layer for initial smoothing and downsampling, followed by several convolutional stages with different kernel sizes to capture spectral features at multiple scales. Adaptive average pooling was then applied to obtain fixed-dimensional feature vectors, followed by a lightweight MLP classification head.

**Figure 9:**
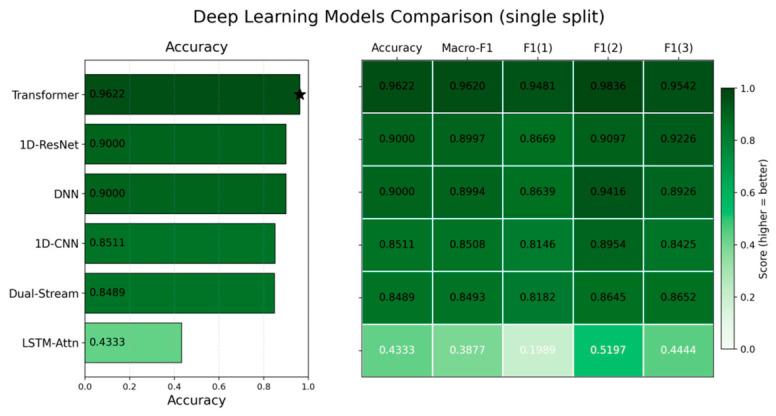
Comparison Plot of Evaluation Metrics. The five-pointed star (★) marks the model achieving the highest classification accuracy.

Under the same data partitioning and evaluation protocol, model-dependent differences were observed among the deep learning architectures. The Transformer achieved the highest classification accuracy of 0.9622, outperforming the other models. Both 1D-ResNet and DNN achieved an accuracy of 0.9000, indicating that residual convolutional feature extraction and fully connected nonlinear mapping were both effective for geographical origin discrimination. The 1D-CNN and Dual-Stream models correctly classified 383 and 382 of the 450 test spectra, corresponding to accuracies of 0.8511 and 0.8489, respectively. Therefore, their performance differed by only one correctly classified spectrum and can be considered essentially comparable under the present test split. Although the Dual-Stream architecture combined a 1D-ResNet branch with a Transformer branch, increased architectural complexity did not necessarily improve generalization. Under the present dataset and training configuration, the two branches may have learned partially redundant representations, while feature fusion and the larger number of trainable parameters may have increased optimization difficulty and overfitting risk. These results indicate that combining local and global feature extractors does not automatically guarantee improved classification performance. The LSTM correctly classified 195 of the 450 test spectra, corresponding to an accuracy of 0.4333. Although this performance remained above the chance level of 0.3333 for three-class classification, it was substantially lower than that of the other deep learning models. In the present implementation, the full spectrum containing 1557 variables was treated as a sequence of 1557 time steps, with one scalar spectral value supplied at each step. No spectral patching, convolutional embedding, or sequence downsampling was applied before recurrent modeling. Processing such a long scalar sequence may have increased optimization difficulty and limited the effective representation of local absorption patterns. Therefore, this result reflects the performance of the specific LSTM configuration evaluated in this study and should not be interpreted as general evidence that LSTM architectures are unsuitable for spectral classification (Figure 9).

The superior performance of the Transformer may be attributed to its ability to model long-range dependencies among spectral bands through the self-attention mechanism. Near-infrared spectra contain not only local absorption-related variations but also global correlations across different spectral regions. Compared with convolution-based models that mainly emphasize local spectral patterns, the Transformer can capture interactions among distant spectral bands, which may improve the discrimination of Fuji apples from different production regions.

### 3.2. Soluble Solid Content Prediction

For the SSC prediction task, PLSR was used as a chemometric baseline and evaluated together with the deep learning regression models using the same training, validation, and test partitioning strategy. The task-specific output layer and optimization objective used for SSC regression are described in Section 2.11. Briefly, each deep learning backbone was adapted for continuous prediction using a single-output linear regression head, without an output softmax activation or cross-entropy loss. The DNN used for SSC regression was implemented as a residual multilayer perceptron containing four MLP blocks and eight hidden fully connected layers, as described in Section 2.9.

To reduce the effects of light scattering, baseline drift, and random noise, the input spectra were preprocessed using standard normal variate transformation and Savitzky–Golay filtering with a window size of 11, a polynomial order of 3, and the first derivative. Feature standardization was subsequently applied. All preprocessing and standardization parameters were estimated using only the training set and were then applied to the validation and test sets to avoid information leakage. The SSC reference values were also standardized using parameters estimated from the training set. After prediction, the model outputs were inverse-transformed to the original °Brix scale for performance evaluation.

**Figure 10:**
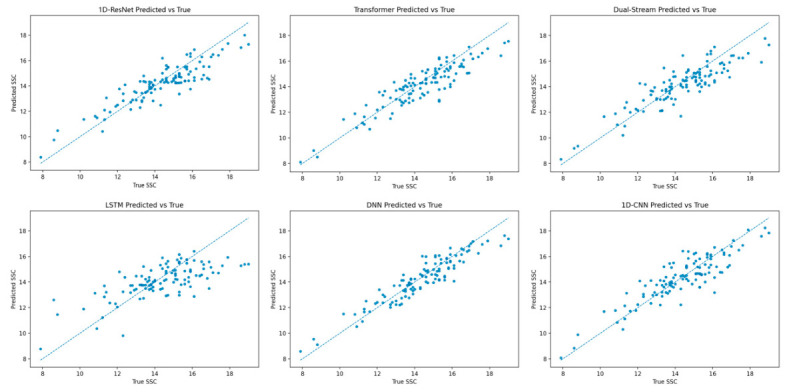
Scatter Plots of Various Models.

**Figure 11:**
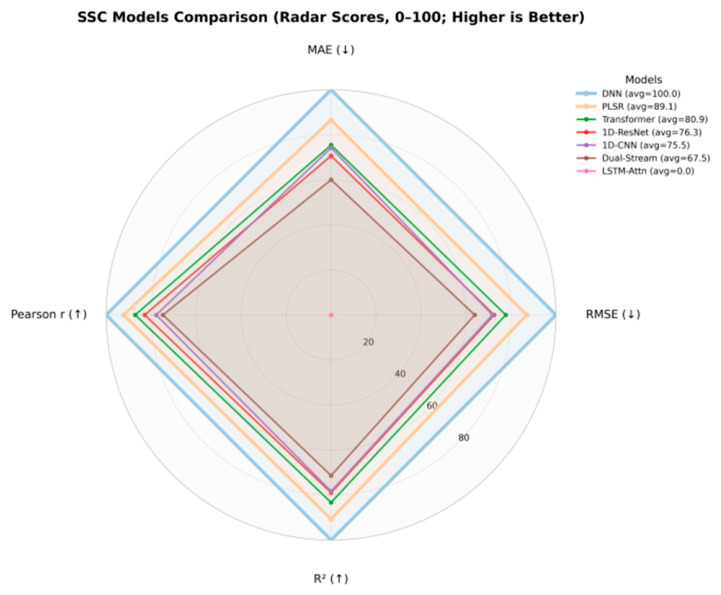
Radar Chart of Comparative Effects.

The predicted-versus-measured SSC values obtained using the different regression models are shown in Figure 10, and their overall performance profiles are compared in Figure 11. Except for the LSTM model, all evaluated deep learning models achieved MAE values below 1 °Brix. This result indicates that most models were able to extract useful SSC-related information from the near-infrared spectra under the present experimental conditions.

Among the evaluated regression models, the DNN achieved the best overall performance, with MAE = 0.5958 °Brix, RMSE = 0.7333 °Brix, R^2^ = 0.8646, and Pearson r = 0.9338. Its predicted values were distributed relatively close to the 45° reference line, indicating good agreement between the predicted and measured SSC values. The PLSR baseline achieved RMSE = 0.8247 °Brix and R^2^ = 0.8288. Compared with PLSR, the DNN showed a lower prediction error and stronger consistency between the predicted and measured SSC trends, indicating that nonlinear spectral–SSC relationships could be effectively captured by the fully connected deep learning model (Figure 11).

In contrast, the LSTM model showed the weakest regression performance, with R^2^ = 0.4793 and Pearson r = 0.7089. The other deep learning architectures showed intermediate prediction performance between the DNN and LSTM models. These differences indicate that model architecture affected the ability to extract quantitative SSC-related information from the one-dimensional spectra, particularly under the relatively limited number of SSC-calibrated samples. The detailed regression performance metrics of PLSR and the deep learning models are summarized in Table 2.

## 4. Discussion

The present study extends previous spectroscopy-based research on apple geographical origin classification by integrating origin authentication with non-destructive soluble solid content (SSC) prediction. Previous studies have demonstrated that apple varieties, orchards, and production regions can be differentiated using compositional profiles, near-infrared spectroscopy, and chemometric or machine learning methods. Guo et al. [1] used polyphenolic profiles combined with PCA and SLDA to classify apple juices according to variety and geographical origin. Li et al. [2], Eisenstecken et al. [3], and Luo et al. [4] further confirmed the feasibility of near-infrared spectroscopy combined with neural networks, support vector machines, PCA-QDA, and wavelength-selection-based discriminant methods for apple variety and origin identification. More recently, Cruz Sanchez et al. [5] evaluated Vis–NIR spectroscopy coupled with machine learning and deep learning methods for classifying apple growing regions, including Fuji apples. Collectively, these studies support the presence of geographical-origin-related information in apple spectral and compositional profiles.

Compared with previous studies that mainly focused on origin or variety classification, the present work evaluated two complementary tasks using the same type of near-infrared spectral information: geographical origin classification, which is associated with traceability and authenticity, and local tissue SSC prediction, which is associated with fruit quality assessment. The main contribution therefore lies in the construction and systematic evaluation of a dual-task spectroscopy framework rather than in the development of a single novel network architecture. Traditional machine learning classifiers and PLSR were included only as contextual baselines, whereas the primary analysis focused on the task-dependent performance of six deep learning model families.

For geographical origin classification, the Transformer achieved the highest test accuracy of 0.9622. This result is consistent with previous evidence that apple spectra contain sufficient information for regional discrimination. However, it should not be interpreted as direct evidence that the Transformer is universally superior to the methods reported in previous studies. Numerical comparisons across studies are limited by differences in cultivar composition, geographical categories, spectral range, measurement instruments, sample size, preprocessing, data partitioning, and validation strategy. In particular, some previous studies used mixed cultivars, manually selected wavelength intervals, imaging-based measurements, or orchard-level validation, whereas the present study used standardized full-spectrum FT-NIR data from Fuji apples collected from three production regions. The principal extension of the present work therefore lies in the dual-task application and systematic architecture comparison rather than in classification accuracy alone.

As reported in Section 3.1, the row-normalized confusion matrices revealed clear class-specific differences among the three geographical origins. Luochuan apples were generally identified more accurately, whereas Xinjiang samples showed greater confusion with the other two origins. This pattern suggests that the Luochuan samples may have exhibited more internally consistent spectral characteristics or greater separation from the other origins under the present experimental conditions. In contrast, partial spectral overlap between Xinjiang and the other regions may have been associated with similarities in maturity, SSC distribution, post-harvest handling, storage history, or scattering-related variation. Because these environmental, compositional, and post-harvest factors were not independently quantified, these explanations should be regarded as plausible interpretations rather than confirmed causes of the observed classification pattern.

The superior classification performance of the Transformer may be associated with its ability to model interactions among separated spectral regions through self-attention. Near-infrared spectra contain both local absorption-related structures and coordinated variations across broader spectral intervals. By dividing the spectrum into patches and learning long-range relationships among them, the Transformer may have captured combinations of spectral information that were useful for origin discrimination. The 1D-ResNet also achieved strong performance, possibly because residual connections improved optimization stability and enabled stacked one-dimensional convolutions to progressively integrate local absorption-related patterns with broader spectral characteristics.

The classification DNN treated each standardized full spectrum as a fixed-dimensional vector and learned nonlinear combinations of all spectral variables through fully connected layers. Its relatively strong performance indicates that the full-spectrum data contained substantial origin-related information even without convolutional or recurrent processing. Nevertheless, the DNN did not achieve the best classification performance, because the Transformer produced a higher test accuracy. The advantage of the DNN was therefore task dependent and was observed primarily in SSC regression rather than in geographical origin classification.

The 1D-CNN and Dual-Stream models produced nearly identical classification results, differing by only one correctly classified test spectrum. Although the Dual-Stream architecture combined local convolutional representations from a 1D-ResNet branch with global attention-based representations from a Transformer branch, hybridization does not inherently guarantee improved performance. Its effectiveness depends on the complementarity of the branch features, the fusion strategy, parameter balance, regularization, and optimization. In the present implementation, concatenating the two branch outputs may have introduced redundant information, increased model capacity, or made optimization more difficult under the available sample size. This explanation remains hypothetical because branch-specific ablation experiments, parameter-matched comparisons, and alternative fusion strategies were not systematically evaluated.

The relatively weak classification performance of the LSTM should be interpreted in relation to its specific input representation and architecture. In the present implementation, the 1557 spectral variables were processed as a sequence of 1557 time steps, with one scalar spectral value supplied at each step. Although a two-layer bidirectional LSTM and an attention mechanism were used, no spectral patching, convolutional embedding, or sequence downsampling was applied before recurrent processing. Modeling such a long scalar sequence may have increased optimization difficulty and reduced the ability of the network to preserve local absorption-related structures. Therefore, the observed result should be regarded as the performance of the evaluated LSTM configuration rather than as general evidence that all recurrent architectures are unsuitable for spectral classification.

The SSC prediction results should also be considered in relation to previous non-destructive apple quality studies. Mendoza et al. [6] combined spectral and image features from hyperspectral scattering data to predict apple firmness and SSC, demonstrating the potential benefit of integrating spectral and spatial information. Fan et al. [7] showed that spectral measurement position can substantially affect the robustness of SSC prediction. Mo et al. [8] used hyperspectral imaging and PLSR to evaluate the spatial distribution of SSC, whereas Lu et al. [9] investigated multispectral imaging for predicting firmness and SSC. These studies employed imaging-based measurements capable of providing spatial information, whereas the present study used point-based FT-NIR diffuse reflectance spectroscopy.

The present study paired each near-infrared spectrum with an SSC reference value obtained from tissue collected at the corresponding sampling position. This position-matched design is consistent with the findings of Fan et al. [7], who emphasized the influence of measurement position on SSC model robustness, and may reduce the mismatch between the spectrally detected tissue volume and the destructive reference measurement. However, unlike hyperspectral imaging, the point-based method used here cannot provide spatial SSC distribution maps. In addition, the predicted values represent local tissue SSC at the measured positions rather than the average SSC of the whole fruit.

Within the present dataset, the residual DNN achieved the best SSC regression performance, with MAE = 0.5958 °Brix, RMSE = 0.7333 °Brix, R^2^ = 0.8646, and Pearson r = 0.9338. It also outperformed the PLSR baseline, which achieved RMSE = 0.8247 °Brix and R^2^ = 0.8288. This within-study improvement suggests that nonlinear spectral–SSC relationships were present in addition to the linear relationships represented by PLSR. Nevertheless, these results should not be directly compared with numerical values reported in previous imaging or spectroscopy studies because of differences in spectral range, sample number, reference-value definition, tissue sampling depth, preprocessing, and validation strategy. The result therefore supports the suitability of the residual DNN for the present dataset but does not establish universal superiority over previously reported models.

The strong regression performance of the residual DNN may be related to the characteristics of the SSC dataset and its task-specific preprocessing. The regression dataset contained 750 SSC-calibrated spectra. Before modeling, the spectra were processed using standard normal variate transformation, Savitzky–Golay first-derivative filtering, and training-set-based feature standardization. These procedures may have reduced scattering effects, baseline variation, and irrelevant spectral variability, allowing the DNN to learn a global nonlinear relationship between the full spectrum and SSC. The residual multilayer perceptron also provided substantial nonlinear fitting capacity while maintaining relatively direct gradient propagation. Under the limited calibration sample size, this structure may have provided a favorable balance among model capacity, regularization, optimization stability, and generalization.

This interpretation should nevertheless be treated cautiously. The present study did not include parameter-matched comparisons, comprehensive architecture ablation experiments, or learning-curve analysis. Therefore, the superior DNN result cannot be conclusively attributed to a single structural property. More complex architectures may require larger calibration datasets, stronger regularization, or more extensive task-specific optimization to fully exploit their representational capacity. The fact that several deep learning models did not consistently outperform PLSR further indicates that increased architectural complexity does not necessarily improve quantitative prediction when the number of calibrated samples is limited.

The relatively weak SSC regression performance of the LSTM, with R^2^ = 0.4793 and Pearson r = 0.7089, similarly suggests that the evaluated recurrent configuration was not well matched to the present regression representation. Local tissue heterogeneity, uncertainty in the destructive SSC reference measurements, and the limited number of calibrated spectra may also have contributed to the prediction errors observed for all models. Consequently, the model rankings reported here should be interpreted within the present dataset, preprocessing procedures, and training configuration rather than as universal rankings of the evaluated architectures.

The interpretation of the SSC results should also consider the physical sampling depth of diffuse reflectance near-infrared spectroscopy. The detected spectral information mainly originated from the peel and shallow flesh tissue near the fruit surface rather than from the entire internal volume of the apple. The corresponding SSC reference values were therefore obtained from tissue collected at the same sampling positions. Accordingly, the regression results represent a non-destructive estimation of local tissue SSC rather than a direct prediction of whole-fruit average SSC. This distinction defines an important application boundary of the proposed approach.

Several limitations of the present study should be acknowledged. First, alternative preprocessing strategies were not systematically compared. Variable-wise standardization was used for geographical origin classification, whereas SNV and Savitzky–Golay first-derivative preprocessing were applied for SSC regression. Controlled preprocessing ablation experiments are needed to evaluate the individual contributions of SNV, MSC, smoothing, derivative transformation, and their combinations. In the present study, all spectral acquisition and SSC measurements were conducted under the same indoor laboratory conditions, with a recorded laboratory temperature of 28 °C. Although this ensured consistency within the present experiment, the effects of different measurement temperatures on SSC reference determination, spectral responses, and spectral-model robustness should be further evaluated under explicitly controlled conditions.

Second, multiple spectra were collected from each fruit, and the present data partitioning was performed at the spectrum level. Consequently, spectra obtained from the same fruit may have been assigned to different training, validation, and test subsets. Future studies should adopt fruit-level grouped partitioning so that all spectra from the same apple remain within a single subset. Cross-batch, cross-season, and independent external validation should also be conducted to provide stricter assessments of model generalization.

Finally, repeated training with multiple random seeds, parameter-matched model comparisons, learning-curve analysis, branch-specific ablation experiments, and alternative feature-fusion strategies would help clarify the sources of performance differences among the evaluated architectures. Residual error analysis and spectral interpretability methods, including attention analysis and feature-contribution assessment, may further identify spectral regions associated with geographical origin and SSC variation and improve the mechanistic interpretation of the models.

## 5. Conclusions

This study established a dual-task near-infrared spectroscopy framework for geographical origin classification and non-destructive local tissue SSC prediction of Fuji apples. For origin classification, the Transformer achieved the highest test accuracy of 0.9622. For SSC regression, the residual DNN achieved the best performance, with MAE = 0.5958 °Brix, RMSE = 0.7333 °Brix, R^2^ = 0.8646, and Pearson r = 0.9338. These results demonstrate that model performance was task dependent and that greater architectural complexity did not necessarily lead to improved generalization. Overall, near-infrared spectroscopy combined with task-specific deep learning models provides a feasible approach for Fuji apple origin authentication and local tissue quality assessment.

## Figures and Tables

**Table 1:** Computing environment and main training parameters.

Parameter	Value
Operating system	Windows 10
Programming language	Python 3.10
Deep learning framework	PyTorch 2.5.0
GPU	NVIDIA GeForce RTX 4060 Ti
seed	42
batch	64
epochs	300
lr	1 × 10^−3^
weight_decay	1 × 10^−4^
patience	12

**Table 2:** Evaluation Metrics of Models.

Model Name	MAE	RMSE	R^2^	Pearson r
PLSR	0.6630	0.8247	0.8288	0.9165
1D-ResNet	0.7426	0.9270	0.7836	0.8953
Transformer	0.7187	0.8906	0.8003	0.9048
Dual-Stream	0.7959	0.9879	0.7543	0.8770
LSTM	1.0960	1.4380	0.4793	0.7089
DNN	0.5958	0.7333	0.8646	0.9338
1D-CNN	0.7249	0.9327	0.7810	0.8839

## Data Availability

The original contributions presented in this study are included in the article. Further inquiries can be directed to the corresponding author.
